# Microwave Heating and Self-Healing Performance of Asphalt Mixtures Containing Metallic Fibres from Recycled Tyres

**DOI:** 10.3390/ma17235950

**Published:** 2024-12-05

**Authors:** Jose Norambuena-Contreras, Jose L. Concha, María J. Varela, Laura Trigos, Lily Poulikakos, Alvaro González, Martín Arraigada

**Affiliations:** 1 Materials and Manufacturing Research Institute, Department of Civil Engineering, Faculty of Science and Engineering, Swansea University, Swansea SA1 8EN, UK; j.norambuena@swansea.ac.uk; 2 LabMAT, Department of Civil and Environmental Engineering, University of Bío-Bío, Concepción 4051381, Chile; jlconcha@ubiobio.cl (J.L.C.); mvarela@ubiobio.cl (M.J.V.); 3 Department of Geology and Geochemistry, Faculty of Sciences, Universidad Autónoma de Madrid, c/Francisco Tomás y Valiente 7, 28049 Madrid, Spain; laura.trigos@uam.es; 4 Empa, Swiss Federal Laboratories for Materials Science and Technology, Überlandstrasse 129, 8600 Dübendorf, Switzerland; lily.poulikakos@empa.ch; 5 Department of Construction Engineering and Management, School of Engineering, Pontificia Universidad Católica de Chile, Avenida Vicuña Mackenna, Santiago 4860, Chile; algonzav@uc.cl

**Keywords:** self-healing, asphalt mixture, metallic fibres, microwave heating

## Abstract

This study investigates how recycled metal fibres from End-of-Life Tyres (ELTs) affect both microwave heating efficiency and crack healing properties in dense asphalt mixtures. The aim is to improve tyre recyclability by using their fibres in asphalt and exploring their self-healing potential with microwave heating. To achieve this, four dense asphalt mixture designs were studied in the laboratory. Each mixture used the same aggregate gradation and bitumen content, but with three different percentages of metallic fibres by binder volume (i.e., 1.5%, 2.5%, and 3.5%), along with an asphalt mixture without fibres serving as a reference material. The microwave heating properties of the asphalt mixtures and their individual components (i.e., aggregates and bitumen) were measured at six different heating times, ranging from 10 to 60 s. Based on the microwave heating results, the cracking and subsequent self-healing properties of the mixtures were evaluated by exposing them to microwave radiation at three heating times: 30, 40, and 50 s. The main results indicated that adding metallic fibres to facilitate microwave heating of the asphalt mixture is unnecessary because healing can be triggered predominately through the aggregates used. Unlike previous studies, it was observed that the healing level of asphalt mixtures, both with and without metallic fibres, increases with the accumulation of crack-healing cycles. Finally, it was determined that the advised microwave heating time for laboratory-sized mixtures, with or without fibres, is 40 s.

## 1. Introduction

**Figure 1:**
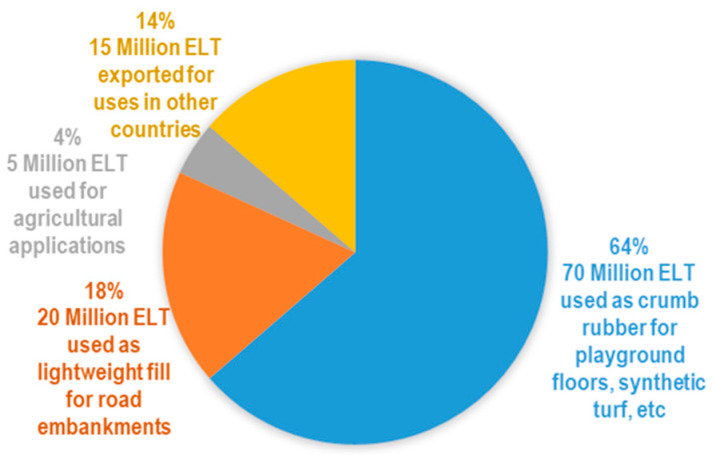
Use of the 110 million tyres processed by the recycling industry each year in the USA, adapted from [8].

The continuous development of the automotive industry generates a high demand for tyre production worldwide. It is estimated that around 2.9 billion tyres are produced worldwide each year, of which a significant part ends up as End-of-Life Tyres (ELTs) in landfills [1]. Globally, ELTs are growing due to the increase in vehicles and traffic [2], and the improper disposal of this waste has become a major environmental problem. According to the European Tyre & Rubber Manufacturers Association (ETRMA), around 3.5 million tons of ELTs were generated in 2019 alone [3], whereas in China and the USA, the yearly disposal reached 14.6 tons and 5.2 tons, respectively [4]. These numbers underscore the need for sustainable disposal solutions. The accumulation of ELTs in landfills or open-air dumps increases the possibility of fire, releases toxic gases, and promotes the proliferation of rodents, insects, and other sources of infection [1,5]. Implementing policies such as the extended producer responsibility has positively contributed to reducing ELT disposal and to increasing innovative use in developed countries [1,6]. Despite this, it is still necessary to make more efforts to reuse ELTs and their by-products (i.e., metallic and textile fibres or rubber powder, among others) in novel solutions for civil engineering. Currently, ELTs and their by-products are used in some countries as fuel for cement kilns, paper mills, or power plants, i.e., energy recovery obtained through thermochemical processes including pyrolysis and thermolysis [2]. Rubber from ELTs is also used in playground and stadium flooring, shock-absorbing mats, paving blocks, roofing materials, etc. [2]. Rubber has also been used as a mechanical reinforcement in asphalt mixtures [7], partially replacing their size-aggregate gradation. As shown in Figure 1, although the recyclability of ELT is nearly complete, their reuse is primarily focused on rubber applications.

ELTs also contain metallic fibres that, unlike rubber, are typically not recycled and are often disposed of in landfills [9,10]. These fibres are obtained as a result of the separation of the different tyre components though tyre shredding, pyrolysis, and cryogenic reduction, followed by a separation though an electromagnetic procedure [11]. However, several studies have explored the potential reuse of these waste fibres in road construction materials. For example, Gonzalez et al. [1] and Ajam et al. [5] evaluated recycled metallic fibres from ELTs as reinforcement additives to improve asphalt mixtures properties. While other metallic fibres such as steel wool fibres are commercially available and have been shown to improve asphalt mixtures’ mechanical and crack-healing performance [10,11,12,13,14,15,16], the recycling of metallic fibres from ELTs offers an environmentally beneficial alternative that has yet to be fully explored.

Bitumen-based pavements possess intrinsic self-healing properties, allowing cracks in a road to close naturally when the temperature rises or during rest periods in the absence of traffic [12,14,16,17,18]. This is because the viscosity of bitumen decreases as the temperature increases. When bitumen reaches a temperature of approximately 30–70 °C, it reduces its viscosity, flowing and autonomously sealing via capillary action the micro-cracks in the asphalt pavement [12,14,15,16,17,18,19,20]. Nonetheless, this natural healing process may require several weeks of constant high temperatures and no loads to restore the original properties of the pavement, which is practically unattainable due to traffic flow and weather conditions. For this reason, numerous researchers [7,12,13,14,15,16,17,18,19,20,21,22,23,24,25,26,27,28,29,30,31] have proposed the use of external heating technologies to accelerate the extrinsic self-healing process of asphalt pavements. These include induction heating [5,15,23,24,25,26,27], infrared heating [28], and microwave heating [12,13,14,15,16,18,19,20,21,26,27,28,29,30,31].

Among these technologies, microwave heating has emerged as one of the most promising electromagnetic heating methods. Microwave energy presents several advantages, such as rapid heating rates, brief curing times, the absence of atmospheric emissions or liquid pollutants, selective material heating, and immediate and precise electronic control over both applied energy and temperatures [23,27,28]. Nevertheless, to understand how microwave heating (dielectric heating) works, it is necessary to grasp its interactions with matter. Microwaves are non-ionising electromagnetic radiation with frequencies ranging from 300 MHz to 300 GHz and wavelengths from 1 mm to 1 m [12,14,15,16,27,28]. They generate heat by making molecules oscillate, rotate, or vibrate [13,15,16,19,27,28], transferring energy to particles. This type of heating by radiation is known as volumetric heating [19,20,27]. Based on their characteristics, microwave-sensitive materials are generally classified into three groups: (a) insulating or transparent, (b) conductive or metallic, and (c) absorbing or dielectric [19,20,28].

Despite its benefits, microwave heating could pose some limitations due to the dielectric properties of the materials. Not all media respond well to dielectric heating, as some are transparent to the dielectric heating process [19,20,28]. In asphalt mixtures, mainly composed of aggregates and bitumen, the latter is almost invisible to dielectric heating. Aggregates, which account for more than 90% by weight of the mixture, are the primary component that is susceptible to dielectric heating, making them responsible for volumetric heating via microwaves [20,27].

Previous studies have demonstrated that adding metallic materials to asphalt mixtures can enhance microwave susceptibility and improve thermal conductivity [13,14,15,19,31]. According to Gallego et al. [19], adding graphene and electric arc furnace slag to asphalt mixtures increases microwave heating efficiency. Similarly, Norambuena-Contreras et al. [14] observed that steel wool fibres improve the heating rate of asphalt mixtures when exposed to microwave radiation. Gallego et al. [29] further suggested that adding steel wool enhances the asphalt mixture’s susceptibility to microwave energy, thereby improving the energy efficiency of the heating process.

On the other hand, the effects of incorporating metallic waste fibres from ELTs on the microwave heating and self-healing properties of asphalt mixtures remain largely unexplored and require further investigation. Moreover, the microwave heating contribution of individual components of the asphalt mixture for crack-healing purposes has not yet been studied, and the effects of fibre distribution and thermal behaviour during microwave heating remain under-researched.

This study therefore examines these aspects, considering conventional Chilean asphalt mixtures. To do this, several asphalt mixture designs were tested in the laboratory with the same aggregate gradation and bitumen content, varying the fibre content. Fibre amounts of 1.5%, 2.5% and 3.5% by volume of bitumen were selected based on previous research on steel wool fibres, which suggested that 4% was the upper limit for achieving various desirable properties [13,14]. A reference mixture without fibre addition was also included for comparison. In a previous study [1], different aspects about the morphology of the metallic fibres and the mechanical properties of the mixtures were studied, revealing their potential for their use in asphalt mixtures with healing purposes.

To provide context for the research, Section 2.1, Section 2.2, Section 2.3 and Section 2.4 of this paper first summarize the findings from previous studies conducted by the authors [1]. Following this, the sections present detailed results on the microwave heating and healing properties of the mixtures and their components. The following sections describe the materials used, their characterization, and the experimental setup employed to assess the crack-healing properties.

## 2. Materials and Methods

### 2.1. Materials

This study used a conventional Chilean dense asphalt mixture with aggregates classified into four factions:

(1) coarse aggregate (CA size 12.5–5 mm),
(2) coarse sand (CS size 2.5–0.32 mm),
(3) fine sand (FS size 0.16–0.08 mm), and
(4) mineral filler (MF size <0.08 mm).

**Figure 2:**
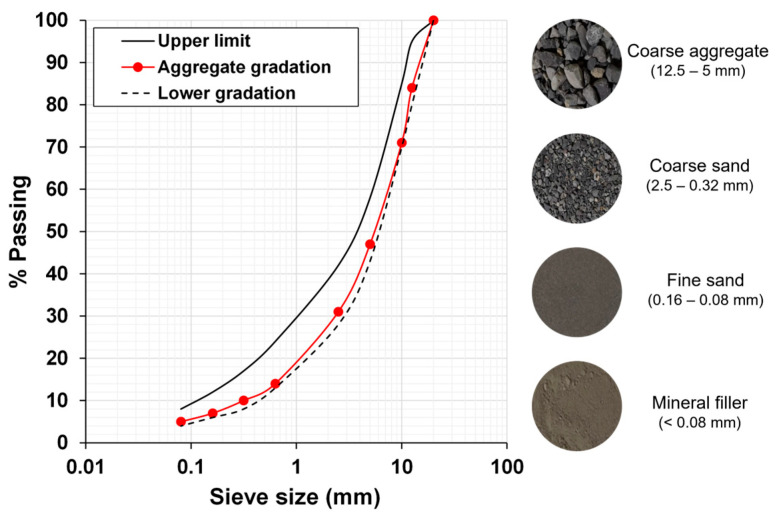
Aggregate gradation used for the dense-graded asphalt mixtures and upper and lower limit according to the Chilean standards [32].

Figure 2 shows the gradation of the aggregates. The limits in the gradation follow the asphalt concrete design Chilean standard [32].

The bitumen used was classified as 50/70 (penetration 66 × 10^−1^ mm at 25 °C and a softening point of 51 °C), and it had a density of 1.039 g/cm^3^. A constant bitumen content of 5.2% by mass of total mixture was used.

In addition, metallic fibres from a plant of Tyre Recycling Solutions S.A. (Préverenges, Switzerland) were added to the asphalt mixture. The fibre material was high-carbon steel with a density of 7.890 < g/cm^3^.

Lastly, three different percentages of metallic fibres were added to the mixtures: 1.5%, 2.5%, and 3.5% per volume of bitumen. These fibre contents were used to guarantee proper mixing during the manufacturing of the asphalt mixtures, as in previous research, higher percentages were related to problems in the compaction and clustering [14]. As a result, four types of asphalt mixtures were prepared: one mixture without fibres (i.e., a reference mixture) and three mixtures with different contents of metallic fibres but using the same aggregate gradation and bitumen content.

The next sections briefly describe the methods used for a physical characterization of the individual mixture components and the resulting mixtures.

### 2.2. Elemental Characterisation of Aggregates and Metallic Fibres

The morphology, material composition, and concentration of fibres added to an asphalt mixture can significantly affect both its mechanical performance and its healing properties. For example, in [14], it was shown that relatively long steel wool fibres tend to form clusters, reducing the bulk density of the mixture and potentially affecting the stress distribution within the asphalt matrix. This clustering could impact the overall performance of the mixture.

**Figure 3:**
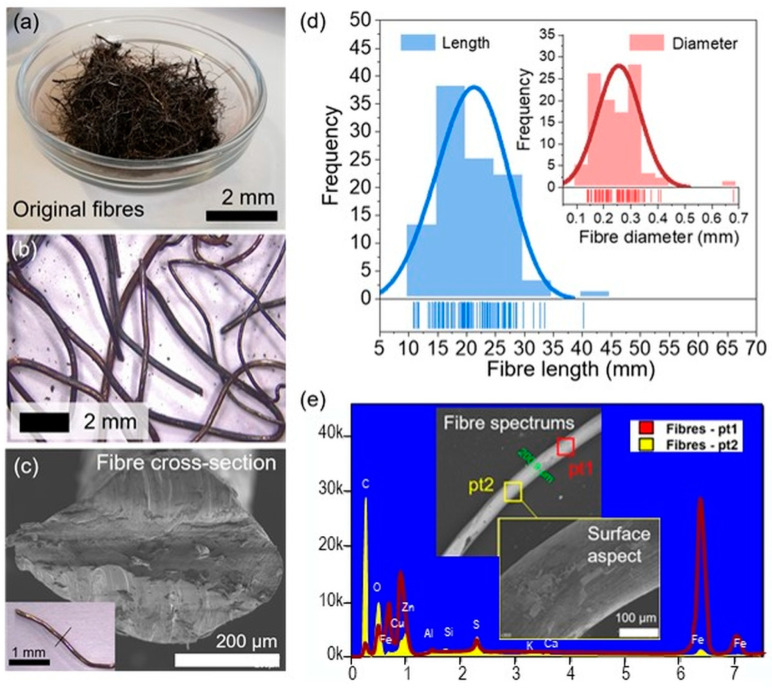
Pictures summarizing the characterization of the fibres (from [1]): (**a**) group of fibres as received; (**b**) optical microscopy image of the cleaned fibres; (**c**) cross-section of the cut fibres with ESEM; (**d**) frequency histogram of the length and diameter of the fibres data with Weibull and Normal fitting, respectively; (**e**) ESEM-EDX analysis before the thermal process.

To better understand the properties of the recycled tyre metal fibres, optical and Environmental Scanning Electron Microscopy (ESEM) tests were conducted. The analysis included the study of the size, surface aspects, and cross-section of the recycled tyre metal fibres. Additionally, the surface aspect and cross-section of the metallic fibres (see Figure 3b,c) were analysed via ESEM-EDX before mixing and compaction. A detailed explanation of the method used and the results can be found in a preceding publication [1]. 

According to these measurements, the average diameter of the fibres was 0.256 mm (Figure 3c,d), while the length observed in a random sample of 100 fibres ranged from 11 to 40 mm (Figure 3b,d). The fibres were used as delivered by the manufacturer, without any adjustments made to reduce their length. As explained in [1], the fibres were thermally treated to reduce their stiffness and length. Figure 3e show an EDX analysis of two points on the fibres before the heating process and the elemental content. Additionally, the aggregates in the asphalt mixture were also characterised using ESEM-EDS analysis using SEM to identify chemical elements that are potentially susceptible to electromagnetic microwave heating.

### 2.3. Manufacturing and Physical Characterisation of Asphalt Mixture Specimens

**Figure 4:**
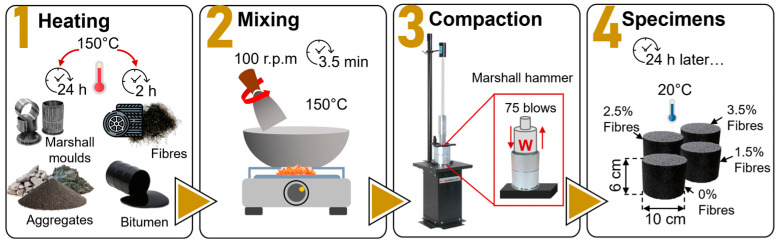
Manufacturing procedure of dense-graded asphalt mixture test specimens with and without metallic fibres. Methodology according to the specifications of Marshall’s design.

To prepare the test asphalt mixture specimens (see Figure 4), materials were mixed in a metallic bowl for 3.5 min at a steady speed of 100 rpm with the mixing temperature kept at 150 °C. Prior to mixing, the aggregates were heated at 150 °C for 24 h, and the bitumen and metallic fibres were preheated at 150 °C for 2 h.

The materials were added to the mixing container in the following order: coarse aggregate, coarse sand, fine sand, metallic fibres, bitumen, and mineral filler. After achieving uniform fibre distribution and complete bitumen coverage, the mixture was poured into a preheated Marshall mould (10 cm in diameter and 6 cm in height) and compacted with 75 blows per side using a Marshall hammer.

Once compacted, the specimens were left to cool at room temperature for 24 h and then mechanically extracted from the mould. The average bulk density and air void contents of the asphalt specimens used in this research, as measured in [1], are reported in Table 1. The table includes a ranking position of each value indicated beside each result in parentheses, with a variable font size. This ranking allows for a quick comparison of performance across various parameters, with higher-ranking values (in larger font) representing the most favourable outcomes for each characteristic. It can be observed that the addition of fibres undesirably reduces the bulk density of the mixtures and increases the air void content. This effect might be caused by the stiffness and size of the fibres, which hinder compaction.

### 2.4. Fibre Distribution Examined Using X-Ray Computed Tomography

In order to investigate if the addition of fibres promote the formation of clusters, several asphalt mixtures—one sample for every fibre concentration—were scanned using X-ray Computed Tomography (CT), as already published in Gonzalez et al. [1]. Cylinders of 40 mm diameter and 40 mm height were cored from the centre of the Marshall asphalt specimens and used as samples for the CT scans. The CT system used was a RayScan500 (RayScan, Meersburg, Germany) equipped with a 300 kV microfocus X-ray source FineTec FORE 300.01Y RT (PerkinElmer, Waltham, MA, USA) and a digital X-ray detector PerkinElmer XRD 1611 CP3 (PerkinElmer, Waltham, MA, USA). The samples were scanned at X-ray source settings of 290 kV/490 µA. A total number of 1260 X-ray projections were acquired with an integration time of 999 ms and a detector pixel binning of 2. The source-detector distance of the scanner was set to 1534.9 mm, and the source-object distance was 445.5 mm. After acquiring the CTs, three-dimensional grayscale images were segmented to identify the individual fibres’ location and volume. Segmentation is the process of splitting the pixels (or voxels in the case of 3D images) corresponding to the individual materials of the composite. Voxel-based segmentation allows us to accurately quantify the spatial distribution of fibres and detect clustering, which directly affects the mechanical and thermal performance under microwave radiation. This work used the known volume of the fibres as a parameter to validate and select the best segmentation method, as explained in detail in [1]. The volume of the metallic fibres obtained from the segmented 3D images was calculated by counting the voxels assigned to the material.

Additionally, 3D images were divided into eight cubic subspaces with side lengths of 150 voxels (ca. 17.4 mm), equivalent to almost 5.3 cm^3^. The volume of the fibres was obtained for each of the sub-volumes. The percentage of fibres in each cubic subspace was compared to evaluate the spatial fibre distribution and possible clusters caused during the asphalt mixing process.

Having characterized the physical properties of the asphalt mixtures with and without fibres and their components, the next sections examines their thermal behaviour and crack healing capabilities under microwave heating.

### 2.5. Microwave Heating of Asphalt Mixtures and Their Components

**Figure 5:**
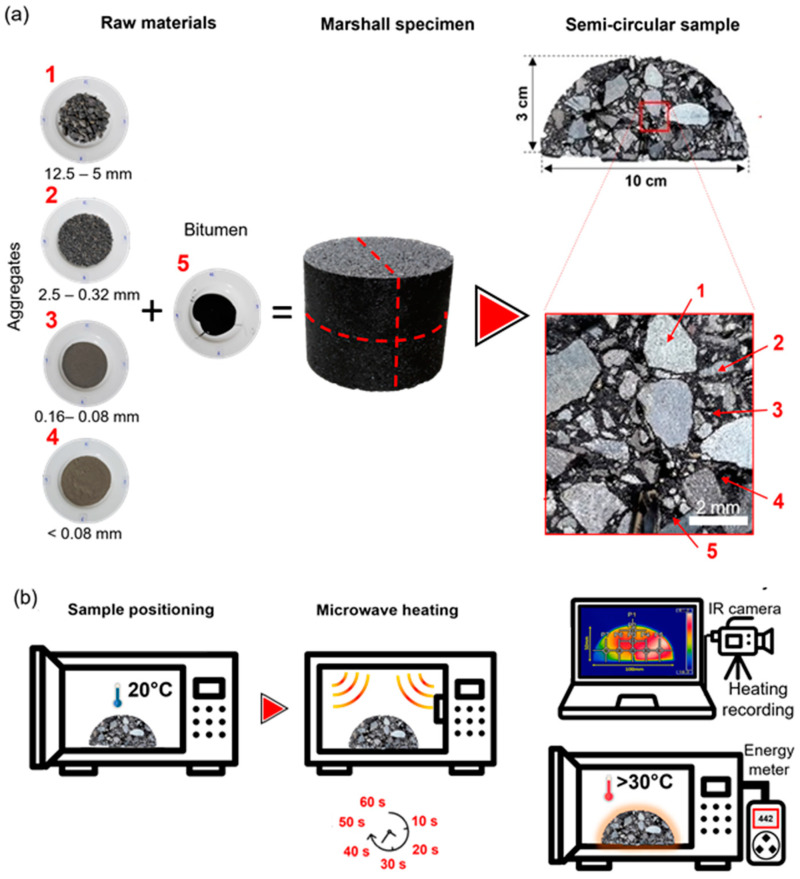
(**a**) Methodology for the heating of mixtures and their components (numbered from 1 to 5) and (**b**) example of microwave heating of asphalt mixtures with semi-circular geometry.

The methodology used to evaluate the microwave heating response of various materials shown with red numbers 1 to 5 in Figure 5, was adapted from previous work by Norambuena-Contreras et al. [13]. As shown in the figure, the experiment involved exposing semi-circular samples of each asphalt mixture design (with and without fibres) and their individual components (aggregates, filler and bitumen) to microwave heating (see Figure 5a,b). The experiment was conducted using a 700 W microwave operating at a frequency of 2.45 GHz. The surface temperatures of these specimens and their components were measured using a PCI PI160 thermographic camera at intervals of 10, 20, 30, 40, 50, and 60 s of microwave exposure. These exposures times were defined based on preliminary tests, which indicated that exceeding 60 s of microwave heating causes the temperature to rise to a level where the specimens become prone to breaking under their own weight.

Four semi-circular asphalt samples, each with a 4 mm thick and 10 mm height notch at the centre, were cut from the Marshall specimens prepared as described in Section 2.3. In addition, the individual components of the asphalt mixture (aggregates = fractions and bitumen, as defined in Section 2.1) were also subjected to the microwave heating. The quantities of each material fraction used are presented in Table 2. These materials were placed in a non-thermally conductive ceramic vessel, transparent to microwaves, and their temperature at the beginning of the heating process was set to 20 °C. To evaluate the effects of aggregate distribution during microwave heating, the materials were randomly mixed in the ceramic vessel and tested in three different configurations. This setup allowed for a more comprehensive assessment of how material arrangement influences microwave heating efficiency.

The energy consumed by the microwave to heat the semicircular mixture samples and their components at various heating times was determined using a Benetech GM89 Digital power meter (Benetech, Shenzhen, China), which measured the power output at certain time interval, allowing for the calculation of the energy used to heat the samples. To quantify the microwave energy at each heating time (10, 20, 30, 40, 50, and 60 s), the power consumed by the microwave for each situation was calculated using the relationship between energy, power, and time, then divided by the mass of the material receiving the microwave exposure:

(1) $$E=\frac{P\times t}{m}$$

where E is the microwave energy consumed per unit time and mass in Wh/kg, P is the actual power consumed by the microwave in W, t is the heating time in h, and m is the mass of the heated sample inside the ceramic vessel in kg.

This equation allows for the calculation of the specific energy input required to heat the asphalt samples and their components, providing a measure of the microwave’s efficiency in transferring energy to the materials. Lower specific energy input values imply that less energy is needed to raise the temperature of the material, leading to reduced heating times for reaching the desired temperature. This not only optimizes the heating process but also improves cost efficiency by minimizing energy consumption. At the same time, the representative temperature reached by the semi-circular samples and also by their individual components was determined as the average of three measurements taken during the experiment, ensuring reliable and consistent data.

### 2.6. Measurements of Crack-Healing Properties of Asphalt Mixtures

The crack-healing properties of the asphalt mixtures, both with and without metallic fibres, were evaluated through three-point bending tests (3-PBT) following microwave heating. To induce a brittle fracture, notched semi-circular samples were preconditioned at 5 °C for 24 h before testing. The tests were conducted using a Controls 34-v1072 universal testing machine (CONTROLS S.p.A., Milan, Italy) equipped with a 50 kN load cell. During the 3-PBT, the notch was aligned vertically in the loading direction to ensure that the initial crack occurred at the notch tip. The procedure for assessing the crack-healing properties of the asphalt samples consisted of the following four steps:

- (S-1) Semi-circular samples were positioned on two support rollers, 80 mm apart. A monotonic vertical load was applied at 0.5 mm/min until failure at the midpoint of the semi-circular arc. As a result, two sections of the semi-circular sample were obtained.
- (S-2) Following the bending test, the cracked samples were left at room temperature for 2 h to reach 20 °C, allowing the surface moisture to fully evaporate.
- (S-3) The two broken sections were realigned and subjected to microwave radiation at three intervals: 30 s, 40 s, and 50 s. Surface temperature changes were monitored using a thermographic camera connected to the PIX Connect software version 2.6.2125.0 (Optris, Berlin, Germany).
- (S-4) After microwave heating, the samples were allowed to cool at room temperature for 2 h followed by conditioning at 5 °C for 24 h. Then, they were subjected to the 3-PBT once more, completing a crack-healing cycle.

**Figure 6:**
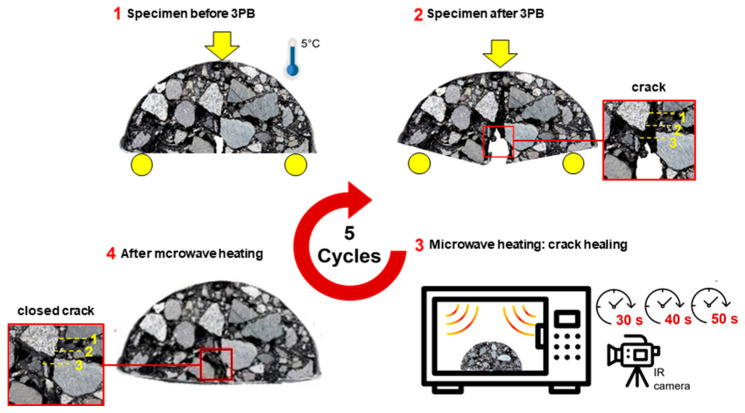
Diagram of the cracking-healing cycle via microwave heating in semi-circular samples.

The diagram in Figure 6 schematically describes steps 1 to 4 which are repeated 5 times. The figure includes three reference yellow dashed lines are used to observe the crack opening in three locations perpedicular to the notch.

**Figure 7:**
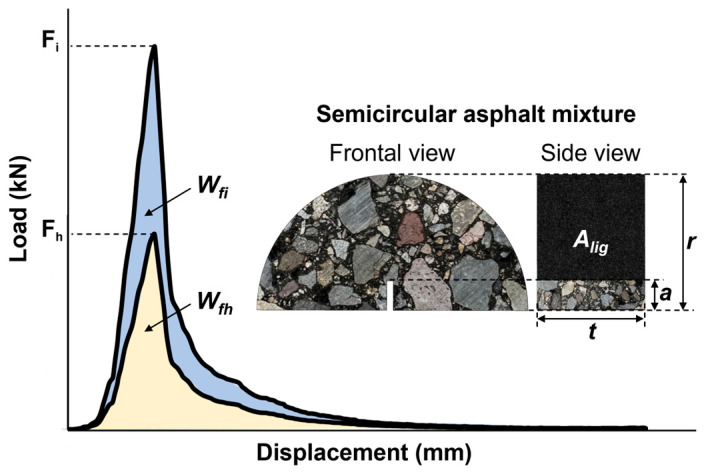
Graphic of peak force and work of fracture based on the load-displacement testing curve.

To quantify the crack-healing properties of the tested asphalt mixtures, two healing indices proposed by Pan et al. [33] were used based on the strength recovery and fracture energy (see Figure 7). Both healing indices are calculated following the calculation steps considered in Equations (2) to (6):

First, the Strength Healing Index (SHI) is the strength recovery ratio, calculated as follows:

(2) $$SHI(\%)=\left(\frac{F_{h}}{F_{i}}\right)\times 100$$

where $F_{i}$ is the maximum initial force recorded by the sample before healing in kN, and $F_{h}$ is the maximum force recorded by the same test sample after the healing process in kN.

Second, the Energy Healing Index (EHI) is the fracture energy ratio, calculated as follows:

(3) $$EHI(\%)=\left(\frac{E_{h}}{E_{i}}\right)\times 100$$

where $E_{i}$ and $E_{h}$ are the fracture energies in J/m^2^ before and after healing, respectively, and are defined as follows:

(4) $$E_{i-h}=\frac{W_{fi-h}}{A_{lig}}$$

In Equation (4), the work of fracture ($W_{fi-h}$) before and after the healing and the ligament area ($A_{lig}$) are determined by Equations (5) and (6), respectively:

(5) $$W_{fi-h}=\sum_{i}^{n}\frac{1}{2}(F_{i+1}+F_{i})(d_{i+1}-d_{i})$$

where $F_{i}$ and $F_{i+1}$ represent the applied load at i and i + 1 load step application in kN, respectively, and $d_{i}$ and $d_{i+1}$ are the displacement at the i-h and i + 1 position in m.

(6) $$A_{lig}=\left(r-a\right)t$$

where r, a, and t are the radius, notch length, and thickness of the test sample in m, respectively.

Finally, to quantify the efficiency of the healing process, five cracking-healing cycles were carried out in the same asphalt test samples, both without fibres and with 2.5% fibre content. The representative healing was determined as the average of three measurements.

### 2.7. Statistical Analysis of the Measured Variables

A statistical analysis of variance (ANOVA) was used in this study to assess whether there were significant differences between the average values obtained for the reference mixture and those for the mixtures with metallic fibres. ANOVA helps determine whether the means of different groups (in this case, results obtained from mixtures with and without fibres) are statistically different from each other.

In this research, the authors selected a confidence interval of 95% to either accept or reject the null hypothesis, which asserts that the fibres do not affect the heating and healing performance. This corresponds to a *p*-value threshold of less than 0.05. To further investigate significant differences among the groups, Tukey’s test was applied as a post hoc analysis. This test provides a detailed examination of pairwise comparisons, allowing for a more detailed understanding of how the presence of metallic fibres affects the performance of the asphalt mixtures.

## 3. Results and Discussion

### 3.1. Thermal Contribution of Asphalt Mixture Components Under Microwave Heating

**Figure 8:**
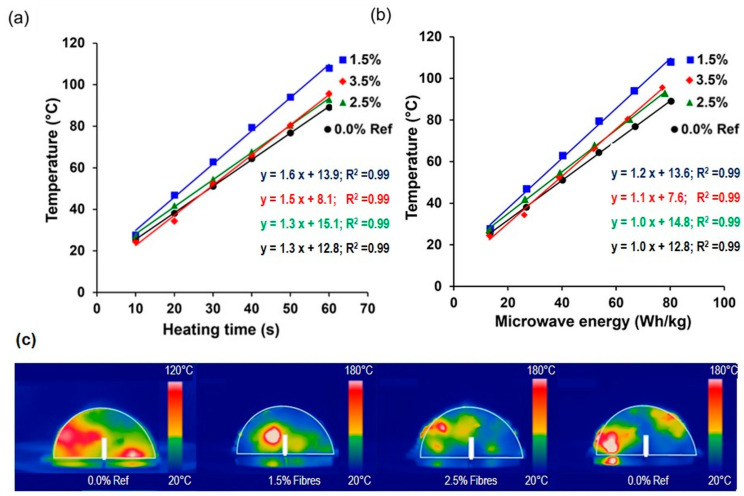
Results of microwave heating of asphalt mixtures with and without metallic fibres: (**a**) average surface temperature reached, (**b**) energy consumed versus surface temperature reached, and (**c**) thermographic images of the surface temperature recorded at a heating time of 60 s.

Figure 8a shows the average surface temperature of the asphalt mixtures with and without fibres obtained from the thermographic images vs. heating time. Each colour represents a different fibre content. The results indicate a direct relationship between microwave heating time and surface temperature, with higher fibre contents leading to faster heating. The heating evolution over time can be characterised by the heating speed of each test sample, as shown by the slope of each linear regression curve in Figure 8a. It is seen that the heating speed of the asphalt mixtures varies with the concentration of fibres, so mixtures with fibre contents of 0.0%, 1.5%, 2.5%, and 3.5% resulted in heating speed values of 1.28 °C/s, 1.60 °C/s, 1.31 °C/s, and 1.45 °C/s, respectively. Consequently, at the end of the heating process, the sample with 1.5% fibre content reached the highest surface temperature, followed by the samples with 3.5%, 2.5%, and 0.0% with 107.9 °C, 95.7 °C, 92.8 °C, and 89.1 °C, respectively. The improved heat distribution in the 1.5% fibre content sample is likely due to a more uniform dispersion of the fibres, avoiding the formation of clusters and therefore preventing thermal hotspots, which enhances overall conductivity.

Similarly, Figure 8b shows the temperature increase as a function of the microwave energy required to heat each sample. The results show a linear relationship between temperature and energy. The slope of the regression line (in °C/(Wh/kg)) indicates the material’s ability to be heated via microwave radiation. A steeper slope implies a higher heating efficiency, meaning that the material heats up more quickly and efficiently. It is observed that the addition of fibres increased the heating efficiency of the asphalt mixture. The heating efficiencies from highest to lowest were 1.20, 1.14, 1.01, and 0.95 °C/(Wh/kg) for samples with 1.5%, 3.5%, 2.5%, and 0.0% fibre contents, respectively.

From Figure 8b, it is also possible to determine the energy required for the asphalt mixtures with and without fibres for self-healing purposes. For this purpose, a previous work carried out by Trigos et al. [20] stated that a temperature of 70 °C for dense asphalt mixtures with self-healing purposes is optimal. By replacing the healing temperature on each of the regression curves in Figure 8b, the microwave energy required to heat the asphalt mixtures with 0.0%, 1.5%, 2.5%, and 3.5% fibre contents was 58, 45, 54, and 55 Wh/kg, respectively. Based on these results, mixtures with fibres need less heating energy than samples without fibres to reach the healing temperature. This result may be due to the propagation of microwave waves (function of microwave power) with higher or lower efficiency depending on the material to be heated. Consequently, for asphalt self-healing purposes, mixtures with fibres demand less energy because the fibres allow for better heat propagation. To provide a clearer overview, Table 3 summarizes the previous results, with ranking positions indicated beside each result in parentheses, as previously described.

Changes in the heating speed and the heating efficiency can be visualised in Figure 8c, which shows representative thermographic images of each asphalt sample after 60 s of microwave radiation. This figure shows that the reference asphalt mixture without fibres presented a more homogeneous distribution of the surface temperature when compared to the samples with metallic fibres. The addition of metallic fibres resulted in a non-uniform surface heating distribution, with hot spots attributed to the presence of the highly thermal conductive additive. This analysis suggests that surface temperature variations and heating efficiency in the asphalt samples can be strongly influenced by the presence of a highly thermally conductive material (i.e., long metallic fibres) and its spatial distribution inside the asphalt mixture.

**Figure 9:**
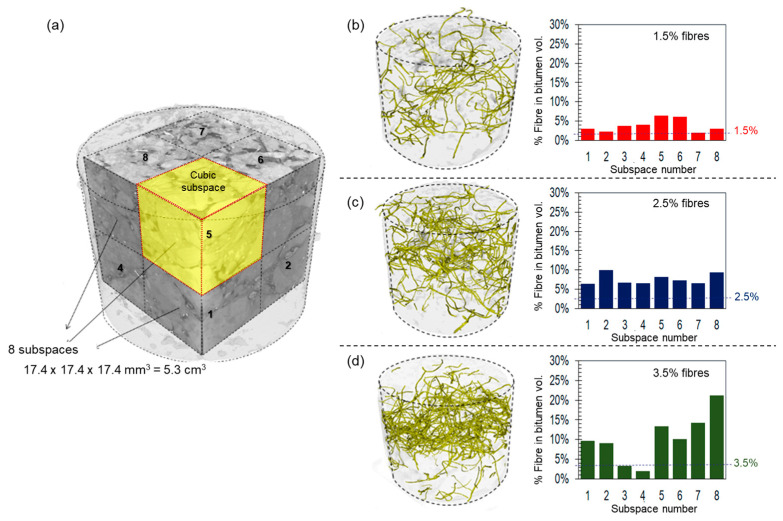
(**a**) On the left side, a view of the cubic sub-spaces used to quantify the spatial distribution of fibres into the asphalt mixture samples. On the right, the 3D CT-Scan of the spatial fibre distribution and a chart with the percentage of fibres in the bitumen volume for each subspace. For asphalt specimens containing (**b**) 1.5%, (**c**) 2.5%, and (**d**) 3.5% fibres (modified from González et al. [1]).

To clarify the observed non-uniform distribution of heat in the specimens with fibres, Figure 9a illustrates the division of the 40 mm diameter cores of eight subspaces, as defined in Section 2.4. These cores were used to investigate the effects of spatial fibre distribution within the samples. On the right side of Figure 9, 3D CT-scan reconstructions display the fibres inside the cylindrical samples, along with bar plots showing the fibre volume in each of the eight subspaces for fibre concentrations of (b) 1.5%, (c) 2.5%, and (d) 3.5% by volume of bitumen. Similar bar heights indicate a homogenous distribution of the fibres. Conversely, poor fibre distribution concentrates the fibres in specific spots, forming clusters (i.e., fibre aggregation inside the asphalt mixture sample), as indicated by significant differences in the bars, with the highest values representing clusters. Consequently, it can be observed that asphalt mixtures containing 2.5% and 3.5% fibre contents have the highest fibre clusters (see Figure 9c,d), which results in localised heating in some areas of the samples (see Figure 8c). In short, the samples with a better spatial distribution of metallic fibres into the asphalt matrix (fibre content of 1.5%) had higher temperatures due to a better heat-control distribution inside the asphalt mixtures. Fibre clustering, observed in mixtures with higher fibre contents, could hinder uniform healing and lead to uneven pavement wear, suggesting that lower fibre concentrations may be more viable for real-world applications.

To better understand the behaviour of the mixtures under microwave heating, each constituent (i.e., bitumen, aggregate and metallic fibres) were individually analysed. For the aggregates, the elemental composition was determined using ESEM-EDX analysis. Due to sample size restrictions, three different grain sizes were analysed:

(i) 12.5–0.32 mm,
(ii) 0.16–0.08 mm, and
(iii) <0.08 mm (filler).

**Figure 10:**
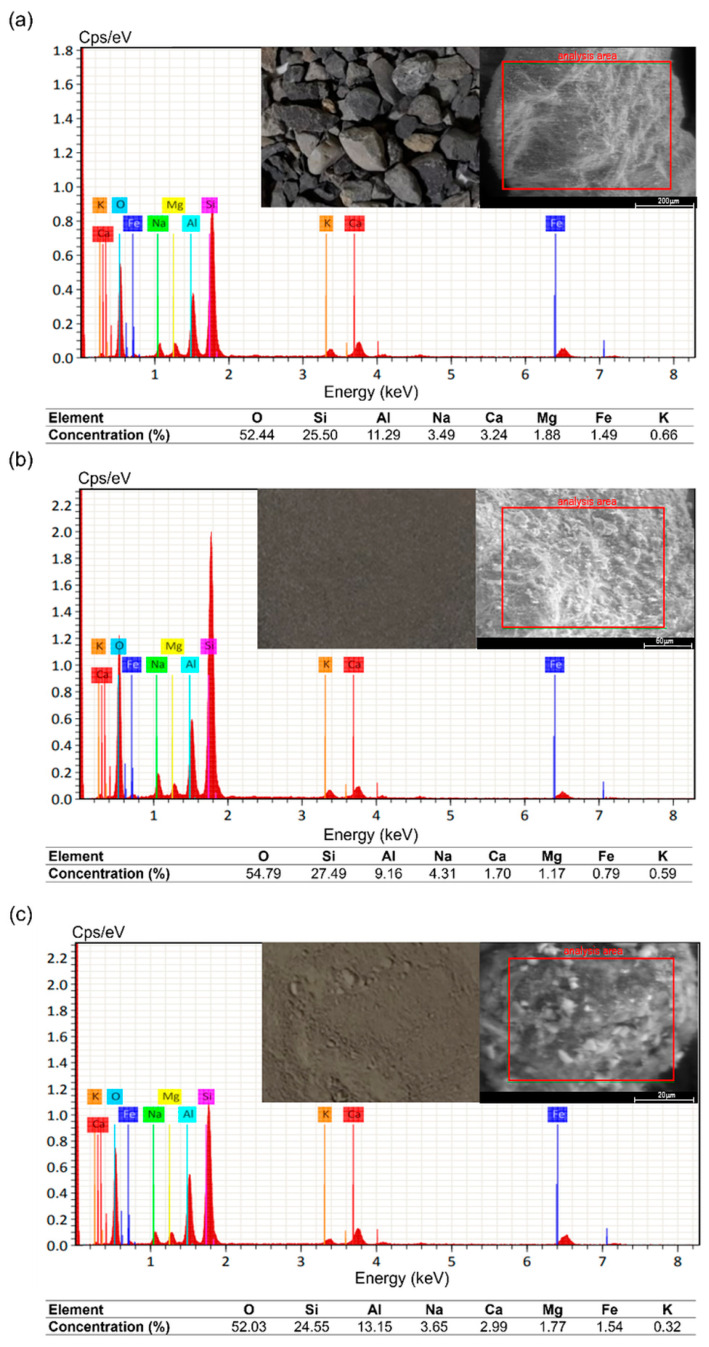
ESEM-EDS elemental analysis of the aggregates depending on their size: (**a**) 12.5–0.32 mm (gravel and coarse sand), (**b**) 0.16–0.08 mm (fine sand), and (**c**) <0.08 mm (filler).

As shown in Figure 10, the ESEM-EDX analysis of the three sizes does not present significant variations between them, ruling out the influence of chemical composition on the differential heating of the asphalt mixtures. Overall, the aggregates contain traces of silicon (Si), aluminium (Al) and iron (Fe), which contributed to the volumetric heating of the aggregates inside the asphalt mixes.

**Figure 11:**
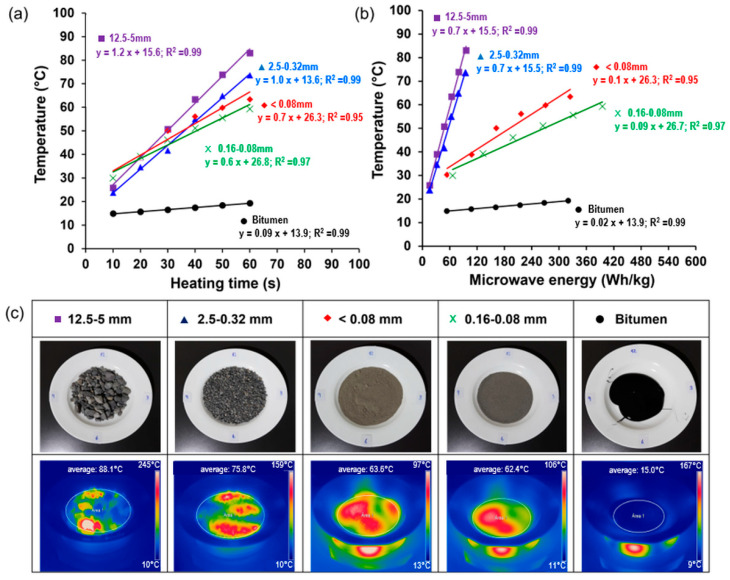
Results of microwave heating of asphalt mixture components: (**a**) average surface temperature reached; (**b**) energy consumed versus surface temperature reached; (**c**) thermographic images recorded at a heating time of 60 s.

The microwave heating and the energy consumption of the four different aggregates gradations and the bitumen (see Table 2) were investigated. The results and the thermographic images obtained after 60 s of microwave irradiation are shown in Figure 11. As illustrated in Figure 11a, the aggregates of the three different sizes reached average temperature values ranging between 60 °C and 80 °C after 60 s of microwave heating. Although larger aggregates reached higher temperatures, these differences are not statistically significant between the four samples studied. Nonetheless, when the heating of the aggregates with bitumen are compared, significant differences are observed, as presented in the Tukey HSD mean comparisons in Table 4. This is because bitumen, a thermoplastic polymer material, has a very low heating rate with an inert behaviour to microwave heating. This confirms previous findings that stated that bitumen (thermoplastic polymer) is inert or almost inert against microwave radiation [34]. At the same time, in the aggregate components, mineralogy and chemistry affect heating [35]. Finally, additives which promote heating, such as metallic fibres, steel wool fibres, or blast furnace slag, could be added to the asphalt mixture for crack self-healing purposes [13,19]. Focused on the aggregates’ behaviour, other properties, such as the size of the crystals of the minerals that compose them, also affect heating, although to a lesser extent [35].

Regarding energy consumption, significant differences are observed among the different aggregate sizes (see Figure 11b and Table 4). For the 12.5–5 mm (coarse aggregate) and 2.5–0.32 mm (coarse sand) particle sizes, the energy used after 60 s of irradiation was approximately 95 Wh/kg. Additionally, for samples with gradations of fine sand (0.16–0.08 mm), mineral filler (<0.08 mm), and bitumen, the approximate energy use after the 60 s of irradiation was between 320 and 400 Wh/kg.

After performing statistical analyses, two different groups were identified: the first, comprising the samples with lower energy consumption (i.e., sizes of 12.5–5 mm and 2.5–0.32 mm), and the second, including the samples with high energy consumption (i.e., sizes of 0.16–0.08 mm, <0.08 mm, and bitumen).

The observed heating and energy consumption differences can be explained by how microwaves work with materials. As previously analysed, the metallic fibres from ELTs would act as a conductive material with a high heating rate. The bitumen can be classified as a transparent material; meanwhile, the aggregates can be classified as dielectrics or absorbents [30] based on a previous study conducted by Trigos et al. [36]. Following this classification, the response of each of these materials to microwave heating can be visualised in the thermal images in Figure 11c. The type of heating that occurs in materials produced by microwaves is known as volumetric heating [37]. Material properties such as moisture content, dielectric properties, geometry, penetration depth, and composition affect microwave heating [30,36,38].

Regarding moisture content, water can appear in different ways [39]. For example, previous studies show that the moisture found in basalt, granite, and sandstone aggregates enhances microwave heating, facilitating heat transmission [40]. Thus, larger aggregates can contain higher water contents, increasing heating efficiency.

Concerning geometry and depth of penetration, in this specific case, both properties are closely related. The aggregates used in asphalt mixtures have generally sub-rounded or sub-angular shapes, enhancing the heating [36]. Nevertheless, considering different gradations, the geometry can vary, and this can affect heating, since spherical shapes favour the heating of the aggregates [41].

Finally, penetration depth involves the transfer of energy into the material [38]. If the penetration depth is less than the size of the sample, the energy will not be totally transferred to the sample, reducing the energy absorption and at the same time raising the energy consumption [42]. This is evident in the case of fine aggregates (0.16–0.08 mm, i.e., fine sand and <0.08 mm, i.e., mineral filler). Further, aggregates of smaller size are easily affected by the extreme temperature, causing faster cooling since they have a smaller mass with respect to their surface.

### 3.2. Effects of Fibres and Heating Time on Crack Healing

**Figure 12:**
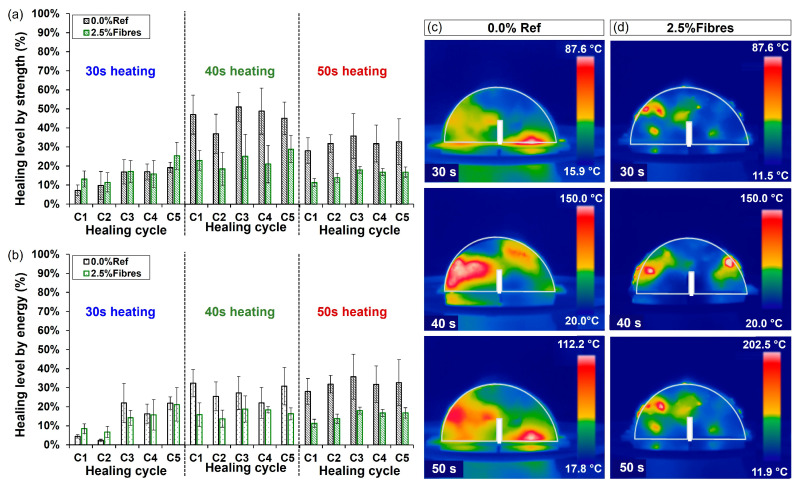
Healing level of asphalt mixtures with x% fibres under (**a**) strength and (**b**) energy criteria at 30 s, 40 s, and 50 s as a function of the number of crack-healing cycles; thermographic images for asphalt mixtures with (**c**) 0.0% and (**d**) 2.5% metallic fibre addition.

To evaluate the influence of the heating time on the healing levels of the asphalt mixture, Figure 12a,b show the average results of healing levels for asphalt samples without and with 2.5% fibre contents heated for 30 s and 50 s as a function of the number of cycles for both strength and energy criteria. From these figures, it can be observed that the healing levels of the asphalt mixtures:

(i) were higher for the reference asphalt mixture without fibres,
(ii) tended to increase with the number of healing cycles, and
(iii) increased for a heating time of up to 40 s.

For heating times longer than 40 s, the healing levels were maintained (under energy criterion) or even decreased (under strength criterion). The associated thermographic images in Figure 12c,d show that the increase in the heating time allows the mixtures with and without fibres to develop a higher temperature in the cracked zone, contributing to sealing of the cracked area. Particularly, Figure 12a,b show that, for the first 2 cycles at 30 s, the mixtures with metallic fibres resulted in healing levels higher than the reference mixture without fibres. This can be attributed to the better heat conduction capacity of the fibres than the asphalt mixture components [16]. Consequently, when the asphalt mixtures with fibres were heated, microwaves could have direct contact with the metallic additive within the asphalt mixture, resulting in greater and faster heat dissipation from the metallic additive to the asphalt matrix. This causes the bitumen to reach temperatures higher than its softening point (~51 °C), reducing its viscosity to seal the open crack more effectively. Consequently, the healing phenomenon in the fibre-reinforced asphalt mixtures occurred at a lower temperature than a standard test sample without fibres.

Nonetheless, Figure 12a,b show that the mechanical strength and healing levels of the asphalt mixture samples with fibres tended to reduce with the successive healing cycles at higher heating times. This may be due to: (1) the rigidity of the metallic fibres, which reduces its contribution to crack-closure, and (2) the localised overheating and ageing of the bitumen near the metallic fibres, leading to a progressive aging and stiffening of the binder, thus decreasing its ability to flow through the cracks.

In Figure 12a, the results of strength (-S) and energy (-E) self-healing levels of the asphalt mixture samples with and without fibres are shown. This figure presents the results of 40 s heating time as a function of the healing cycle number. Overall, Figure 12a shows that the healing level in the asphalt mixtures:

(i) decreased with the incorporation of metallic fibres;
(ii) was lower for the energy criterion compared to the strength one; and
(iii) did not clearly vary with the healing cycles.

**Figure 13:**
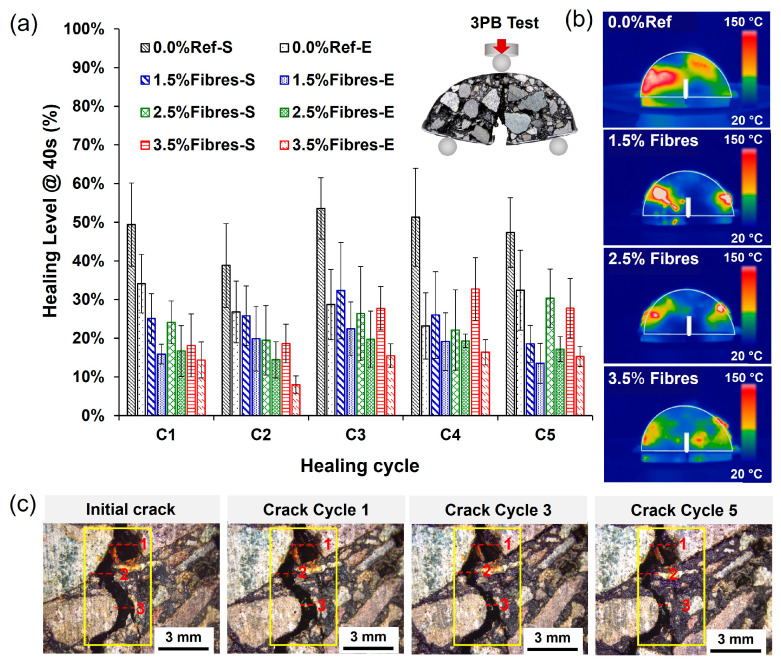
Results of the healing levels reached by the asphalt mixture samples heated for 40 s: (**a**) healing level of samples as a function of the number of crack-healing cycles; (**b**) thermographic images of samples with and without metallic fibres; (**c**) representative optical images of a cracked asphalt sample with metallic fibres before and after the cracking-healing cycles, including three reference (red) lines for comparison purposes. S: strength healing index; E: energy healing index.

Consequently, the overall decrease in the crack-healing level with the addition of fibres can be explained by the heterogeneous distribution of the metallic fibres with a local heating effect (see discussion in Section 3.1). This is supported by the thermographic images in Figure 13b, showing that the mixture without fibres presented a more homogeneous temperature distribution, with values above 70 °C near the fractured zone, facilitating the flow of the bitumen in the damaged zone to close the crack. On the contrary, the fibre-reinforced asphalt mixtures developed temperatures below 40 °C in the fractured area, with hotspots far from the fractured zone. Furthermore, the highly rigid nature of the metallic fibres made the crack closure of the fractured asphalt samples difficult, as exemplified in the Figure 13c (see reference segmented red lines).

In contrast, reduction of the healing values under the energy criterion is explained, since this condition considers the continuous energy absorbed until the fracture of the samples, while the healing based on the strength criterion only considers a discrete point associated to the maximum strength developed by the samples when fractured. Therefore, the healing based on the energy criterion (bars with horizontal lines in Figure 13a) can be a more representative indicator of the crack-healing process measured. Furthermore, the overall lack of a trend in the healing levels with the healing cycles can be explained by the fact that the successive healing cycles using microwave heating can modify the internal microstructure of the asphalt mixtures by repositioning the aggregates within the samples and altering their air void contents, as previously evidenced by Norambuena-Contreras et al. [15]. Finally, based on the previous analysis, it is concluded that the most influential factor in the healing level of the asphalt mixtures was the addition of metallic fibres, decreasing the healing capacity of the mixtures with respect to a reference, regardless of the fibre content and healing cycle.

In summary, the previous analysis shows that the healing level of asphalt mixtures depends on both the heating time and the metal fibre addition. For a practical in situ repair application, it is recommended to heat the mixtures with and without fibres for 40 s, considering that both mixtures registered a similar and stable healing level as the crack-healing cycles increased. This confirms previous studies with similar recommended heating times [16]. Nevertheless, the high stiffness of the metallic fibres from ELTs and their poor distribution within the dense asphalt mixture can reduce the mechanical properties of the mixtures; therefore, their use in onsite asphalt pavements could not be technically attractive to improve their crack-healing behaviour via microwave heating.

## 4. Conclusions and Outlook

This study analysed the effects of recycled metallic fibres from waste tyres on microwave heating. Additionally, this study explored how these fibres influenced the crack-healing properties of dense asphalt mixtures and their components. Based on the analysis of the results, the following conclusions were obtained:

- Key factors affecting healing levels: The main influential factors were the heating time and the addition of metallic fibres, regardless of the fibre content.
- Aggregate grain size and energy consumption: Smaller aggregates required approximately three times more energy to reach the same temperature as larger aggregates. However, there were no significant differences in heating efficiency between the two. Additionally, bitumen was found to be transparent to microwaves.
- Heating time and fibre content on surface temperature: In general, the longer the microwave heating time and the lower the metallic fibre content in the asphalt mixture, the higher the average surface temperature reached by the test samples.
- Heating efficiency: The addition of fibres reduced the required heating energy for the fibre-reinforced mixtures. This positive effect could lower both the environmental footprint and operational costs of asphalt pavement maintenance, aligning with sustainability goals in civil infrastructure.
- Time and fibre influence on healing: Microwave heating facilitated the self-healing of cracked asphalt mixtures, regardless of the presence of metallic fibres. Furthermore, the healing levels of the asphalt mixtures improved with heating times of up to 40 s; this value was selected as the optimal healing time. Longer heating times led to reduced healing levels of the asphalt mixtures with fibres. This can be attributed to changes in the internal structure of the heated samples and potential ageing damage to the bitumen exposed to high temperatures. All healing cycles evaluated in this study achieved similar average healing levels for asphalt mixtures with and without metallic fibres. However, higher amounts of metallic fibres can introduce heterogeneities in the thermal behaviour of the asphalt mixture, creating hot spots that may cause the bitumen to flow away from the fracture zone, thereby affecting the healing process.
- Implications for practical application: Overall, the high stiffness and uneven volumetric distribution of the metallic fibres were found to reduce the mechanical properties of the mixtures. Consequently, in their current form, the use of these fibres in asphalt pavements may not be technically advantageous for enhancing crack-healing behaviour through microwave heating. Furthermore, as this research was conducted in a controlled laboratory environment, additional investigations are needed to scale up and validate these findings for field applications.
- Outlook: Although these findings may discourage the use of ELT recycled metallic fibres in their present form, their potential could improve with advancements in fibre processing techniques to achieve better distribution within the mixture matrix. Future research should therefore explore different fibre processing methods to optimize their performance.

## Figures and Tables

**Table 1:** Physical properties of the asphalt mixture specimens [1].

Fibre Content (%vol. Bitumen)	Bulk Density (g/cm^3^)	Air Void Content (%)
Ref. 0%	2.38 ± 0.20 (1)	4.7 ± 0.43 (1)
1.5%	2.31 ± 0.24 (3)	7.7 ± 0.81 (4)
2.5%	2.33 ± 0.31 (4)	6.1 ± 0.52 (2)
3.5%	2.30 ± 0.26 (2)	7.7 ± 0.71 (3)

**Table 2:** Mass of the fractions of materials of the asphalt mixture tested via microwave heating.

Sample n° (See Figure 5a)	Components of the Mixture	Size (mm)	Mass of Fraction in Marshall Specimen (g)	Testing Mass (g)
1	Coarse aggregate	12.5–5.0	583	200
2	Coarse sand	2.5–0.32	407	200
3	Fine sand	0.16–0.08	52	46
4	Mineral filler	<0.08	58	56
5	Bitumen	-	58	60

**Table 3:** Summary of the results for microwave heating on the asphalt samples.

Fibre Content (%vol. bit.)	Heating Speed (°C/s)	Surface Temp. (°C)	Heating Efficiency °C/(Wh/kg)	Microwave Energy (Wh/kg)
Ref. 0%	1.25 (4)	89.1 (4)	0.95 (4)	58 (4)
1.5%	1.60 (1)	107.9 (1)	1.20 (1)	45 (1)
2.5%	1.31 (3)	92.8 (3)	1.01 (3)	54 (3)
3.5%	1.45 (2)	95.7 (2)	1.14 (2)	55 (2)

**Table 4:** ANOVA *p*-values for microwave heating and energy consumption of the raw materials.

Variable	*p*-Value	Statistically Significant Differences?	Tukey HSD Mean Comparisons
Heating	<0.001	Yes	CA-B; CS-B; FS-B; MF-B
Energy	<0.001	Yes	CA-FS; CA-MF; CA-B CS-FS; CS-MF; CS-B

Notes: CA: coarse aggregate (12.5–5 mm); CS: coarse sand (2.5–0.32 mm); FS: fine sand (0.16–0.08 mm); MF: mineral filler (<0.08 mm); B: bitumen.

## Data Availability

The original contributions presented in the study are included in the article, further inquiries can be directed to the corresponding author.
